# Encapsulation of ɣ-Aminobutyric Acid Compounds Extracted from Germinated Brown Rice by Freeze-Drying Technique

**DOI:** 10.3390/molecules29215119

**Published:** 2024-10-30

**Authors:** Tarinee Nilkamheang, Chanikarn Thanaseelangkoon, Rawinan Sangsue, Sarunya Parisaka, Le Ke Nghiep, Pitchaporn Wanyo, Nitchara Toontom, Kukiat Tudpor

**Affiliations:** 1 Public Health and Environmental Policy in Southeast Asia Research Cluster (PHEP-SEA), Mahasarakham University, Kham Riang, Maha Sarakham 44150, Thailand; tarinee.n@msu.ac.th (T.N.); nitchara.t@msu.ac.th (N.T.); 2 Faculty of Public Health, Mahasarakham University, Kham Riang, Maha Sarakham 44150, Thailand; ninachanikarn777@gmail.com (C.T.); rawinan.02.10.2542@gmail.com (R.S.); aum.sarunya408@gmail.com (S.P.); 3 Vinh Long Department of Health, Vinh Long 85000, Vietnam; lekenghiep@gmail.com; 4 Department of Food Technology, Faculty of Agricultural Technology, Kalasin University, Kalasin 46000, Thailand; pitchaporn.wa@ksu.ac.th

**Keywords:** pigmented rice brans, in vitro simulated digestion, phenolic compounds, antioxidants, bioaccessibility

## Abstract

Gamma-aminobutyric acid (GABA) from plants has several bioactivities, such as neurotransmission, anti-cancer cell proliferation, and blood pressure control. Its bioactivities vary when exposed to pH, heat, and ultraviolet. This study analyzed the protective effect of the GABA encapsulation technique using gum arabic (GA) and maltodextrin (MD) and the freeze-drying method. The impact of different ratios of the wall material GA and MD on morphology, GABA content, antioxidant activity, encapsulation efficiency, process yield, and physical properties were analyzed. Results showed that the structure of encapsulated GABA powder was similar to broken glass pieces of various sizes and irregular shapes. The highest GABA content and encapsulation efficiency were, respectively, 90.77 mg/g and 84.36% when using the wall material GA:MD ratio of 2:2. The encapsulated powder’s antioxidant activity was 1.09–1.80 g of encapsulation powder for each formula, which showed no significant difference. GA and MD as the wall material in a 2:2 (*w*/*w*) ratio showed the lowest bulk density. The high amount of MD showed the highest Hausner ratio (HR), and Carr’s index (CI) showed high encapsulation efficiency and process yield. The stability of encapsulated GABA powder can be kept in clear glass with a screw cap at 35 °C for 42 days compared to the non-encapsulated one, which can be preserved for only 18 days under the same condition. In conclusion, this study demonstrated that the freeze-drying process for GABA encapsulation preserved GABA component extracts from brown rice while increasing its potential beneficial properties. Using a wall material GA:MD ratio of 2:2 resulted in the maximum GABA content, solubility, and encapsulation efficiency while having the lowest bulk density.

## 1. Introduction

Gamma-aminobutyric acid (GABA), an amino acid produced through glutamate decarboxylation, is found in several plants, such as potatoes, germinated soybeans, and brown rice [1,2]. Bioactivities of GABA include inhibiting neurotransmitters, relieving stress, anti-cancer and anti-diabetic properties, and reducing blood pressure [1,3]. According to these properties, it garnered much regard for use in the food and nutraceutical industries. However, GABA is naturally degraded under acidic pH, heat, and UV [4]. Encapsulation encases an inert wall material matrix (encapsulant) around a functionally active core molecule [5]. It has multiple uses in the food industry, such as covering up unwanted color, flavor, or taste, preserving unstable materials, adding more functional and nutritional elements, and releasing encapsulated nutrients at a precise location at a predetermined rate and time [6].

There are many different methods of encapsulation, depending on the types of core molecules and encapsulants, the size of the desired capsules, and other factors, such as resistance to high temperature or physical state [7]. Among several encapsulation techniques, freeze-drying is one of the most valuable processes suitable for molecules sensitive to temperature, oxygen, pH, and light, such as polyphenol, β-carotene, and phycocyanin [8,9,10]. The selection of encapsulants is one factor affecting the capacity of encapsulated particles. There are several encapsulants, such as modified starch, maltodextrin (MD), gum arabic (GA), cyclodextrin, whey protein, etc. MD, the most widely used encapsulant with high solubility and low viscosity at high solids concentration, forms film quickly and creates a barrier from oxygen, with a neutral taste and aroma, and at a low relative cost [11]. Another possible encapsulating agent is GA, which has high-quality emulsifying properties, film-forming capacity, high solubility, low viscosity, and non-toxicity [12]. Several studies showed that mixed GA and MD had more stability than the isolated use of MD and GA [10,13,14]. However, no investigation has been conducted on GABA encapsulation. This study aimed to evaluate the stability of encapsulated GABA compounds extracted from germinated brown rice using the freeze-drying technique using MD and GA as wall materials.

## 2. Results and Discussion

### 2.1. GABA Content

Khao Dawk Mali 105 (KDML 105), a jasmine rice variety known for its superior cooking quality, aroma, and softness, is produced in Thailand and exported to global rice trading markets [15]. KDML 105 brown rice contains GABA content at 13.08 mg/g dried weight (Table 1). After germination, the GABA content was more than double that of the non-germinated one. During the germinating process, rice grain was adsorbed with water, causing an increase in free water in the rice grain. After that, enzymes in the rice grain were activated to decompose stored nutrients, such as carbohydrates and proteins [16]. Also, the glutaminase decarboxylase enzyme was induced to change glutamic amino acid to GABA compounds [17]. According to this phenomenon, there was an increase in GABA content in germinated brown rice. The germinated KDML 105 brown rice extract contained a GABA content of 129.12 ± 1.83 mg/mL. The germinated brown rice extract was measured for antioxidant activity, which showed 86.18 ± 2.44 mL.

### 2.2. Morphology of Encapsulated GABA

**Figure 1:**
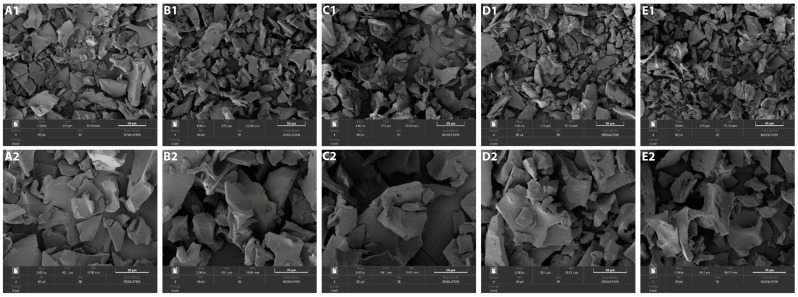
SEM images of encapsulated GABA with different wall material ratios (GA:MD); ratio 0:4 1000× (**A1**), 3000× (**A2**); ratio 1:3 1000× (**B1**), 3000× (**B2**); ratio 2:2 1000× (**C1**), 3000× (**C2**); ratio 3:1 1000× (**D1**), 3000× (**D2**); ratio 4:0 1000× (**E1**), 3000× (**E2**).

GA and MD are the most commonly used wall materials for encapsulation. Their excellent properties include low bulk density and viscosity, forming film easily, creating a barrier from oxygen, and being good at emulsifying. The effect of different wall material GA and MD ratios as 0:4, 1:3, 2:2, 3:1, and 4:0 (GA:MD, *w*/*w*) on morphology, encapsulation efficiency, process yield, and physical properties were analyzed. The morphology of encapsulated GABA was studied based on SEM images, as shown in Figure 1. The structure of encapsulated GABA powder was similar to that of broken glass pieces of various sizes and irregular shapes. A glassy structure with an irregular shape might protect the bioactive compounds against heat and oxygen exposure [10]. The increase of MD as wall material resulted in more porous powder. The large size of freeze-dried particles is due to the low-temperature process and lack of strength to break the frozen surface during drying [18]. The high amount of GA showed a less porous structure, possibly due to increased hygroscopicity. However, the different ratios of wall material GA and MD showed no difference in morphology images by SEM in this study.

### 2.3. Encapsulation Efficiency and Process Yield

The final GABA content of encapsulated GABA using different ratios of wall material GA and MD was found in 54.75–90.77 mg/g of encapsulated GABA powder for each formula (Table 2). The highest GABA content was 90.77 mg/g, which used the wall material GA:MD ratio as 2:2 (*w*/*w*). The encapsulation powder was measured for antioxidant activity, which was found in the range of 1.09–1.80 g of encapsulation powder for each formula, which showed no significant difference (*p* > 0.05). The initial GABA content of 1291.2 mg in 10 mL of GABA extract solution was used, followed by Equation (1) to calculate encapsulation efficiency. The high encapsulation efficiency was found at 84.36%, using the ratio of wall material GA:MD as 2:2 (*w*/*w*), as shown in Figure 1. The decrease and increase of GM content showed less encapsulation efficiency. The using of pure GA as wall material gave higher encapsulation efficiency than using of pure MD, which was found in encapsulated C-phycocyanin extract from Arthrospira by freeze-drying technique [10] and encapsulated *Elsholtzia ciliata* ethanolic extract by freeze-drying technique [19]. However, several studies showed that pure GA and pure MD were less stable of bioactive compounds than mixed GA and MD [10,13,14,20]. The study by Hassain and co-workers showed high efficiency in protecting polyphenol compounds by using pure GD and the mixer of GA and MD as a 1:1 ratio, which was carried out using the freeze method [21].

**Figure 2:**
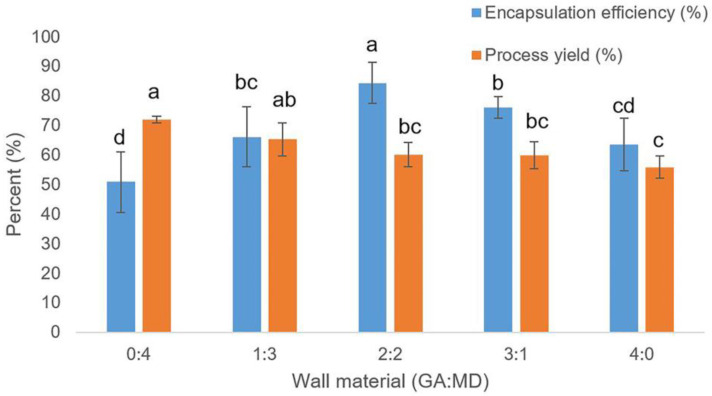
Encapsulation efficiency (%) and process yield (%) of encapsulated GABA with different wall material ratios (GA:MD). Different letters indicate the values with significant differences (*p* ≤ 0.05).

The process yield refers to the solid recovery of encapsulating power amount [22]. Using a high amount of MD was found to have the highest process yield at 72.07%, 1.29 times higher than pure GA, as shown in Figure 2. The decrease in the amount of MD showed a reduction in process yield in the 55.84–65.31% range. Maltodextrin is a drying agent, which increases the bulking agent. The increase in maltodextrin content escalated the glass transition temperature (Tg) value of the powder product, reducing the stickiness inside the drying chamber and resulting in the maximum powder [8].

### 2.4. Physical Properties of Encapsulated GABA Powder

The moisture content of food influences its storage, packaging, and processing [23]. At the same time, water activity plays a significant role in determining quality change and microbial growth or survival as it indicates the amount of free water available for microbial growth and quality change [10]. Solubility is essential in evaluating powders’ re-dissolved effect, influencing their application in an aqueous system [14]. Furthermore, powder particles with low moisture content made their low content denser and decreased their bulk density [24]. The bulk density value would be related to the Hausner ratio and Carr’s index, which showed the powder particle’s flowability. The higher the moisture content, the greater the cohesive forces, resulting in poor flowability. Flowability is an essential factor influencing their application in aqueous systems [25]. According to those data, the excellent quality of powder particles should be low in moisture content, water activity, and bulk density; in contrast, solubility and flowability should be high.

The present study found that a high amount of MD gave high moisture content and water activity (Table 3). The moisture content and water activity of encapsulated GABA compound were 3.79–6.40% and 0.36–0.58, respectively. Data obtained by Sidlagatta and Pudziuvelyte and colleagues corresponded to our study, which showed that the use of pure MD resulted in higher moisture content than using a mixture of MD with other wall materials such as GA and whey protein [19,24]. Adding MD improved the total solid in the feed liquid, and it would be difficult for water molecules to diffuse past the large molecule of MD holding more water within the matrix [24]. Also, using a low-temperature process with freeze-drying results in a smaller pore size and acts as a barrier against sublimation, retaining moisture in freeze-dried powder [19].

The solubility of encapsulation GABA ranges from 80.36 to 90.62% (Table 3). The use of GA and MD at 2:2 *w*/*w* showed the highest solubility. The study of Pudziuvelyte et al. found that using pure MD and a mixture of MD with GA resulted in higher solubility than pure GA [19]. The study of Yu and Lv showed that using MD and GA to encapsulate anthocyanin compounds improved the solubility of anthocyanin compounds due to the high water solubility and better dispersibility of MD and GA [14].

The bulk density of encapsulated GABA was found in 0.47–0.50 g/mL (Table 3). It was observed that using GA and MD as wall material (2:2, *w*/*w*) showed the lowest bulk density, while using GA and MD as wall material (4:0, *w*/*w*) showed the highest bulk density. The high amount of MD decreased in bulk density, but the high amount of GA increased in bulk density. However, the significant analysis found no significant difference (*p* > 0.05). An increase in maltodextrin content led to a decrease in the bulk of lime juice powder, tomato juice powder, and orange powder [26,27]. The high concentration of MD caused reduced bulk density that might have minimized thermoplastic particles from sticking, which might have improved particle volume due to entrapped air [24].

The substance’s flowability is a significant property that influences its handling, transportability, storage, and ability to blend, process, and pack up. Greater Carr’s index (CI) and Hausner ratio (HR) indicate that the powder is more viscous and less able to flow easily [28]. Encapsulated GABA’s flowability was calculated regarding CI and HR, as shown in Table 4. The flowability was excellent if CI and HR were less than 10% and 1.11, respectively. The low CI (11–15%) and HR (1.12–1.18) indicated good flowability. The relatively high CI (16–20%) and HR (1.19–1.25) indicated fair flowability. The very high CI (>31%) and HR (>1.46) indicated very poor flowability. The CI of encapsulated GABA was observed in 29.72–44.56%, and the HR of encapsulated GABA was observed in the range of 1.42–1.82. The high amount of MD showed the highest in CI and HR, indicating poor flowability. The increase in the amount of GA decreased both CI and HR. The high amount of GA showed the lowest in CI and HR, indicating poor flowability. The result was supported by Pandey and Mishra, who showed that encapsulated GABA and probiotic bacteria with a spray drying method by using only MD as the wall material showed a slightly higher CI and HR, possibly due to higher moisture content, causing particles to agglomerate [3]. Pudziuvelyte et al. showed that encapsulating *Elsholtzia ciliata* ethanolic extract with the freeze-drying technique using only MD as the wall material showed lower CI and HR than GA. However, CI and HR increased when using their mixture as the wall material [19]. The results of this study indicated that using MD and GA can improve the flowability of encapsulated GABA.

### 2.5. Stability of Encapsulated GABA Powder

**Figure 3:**
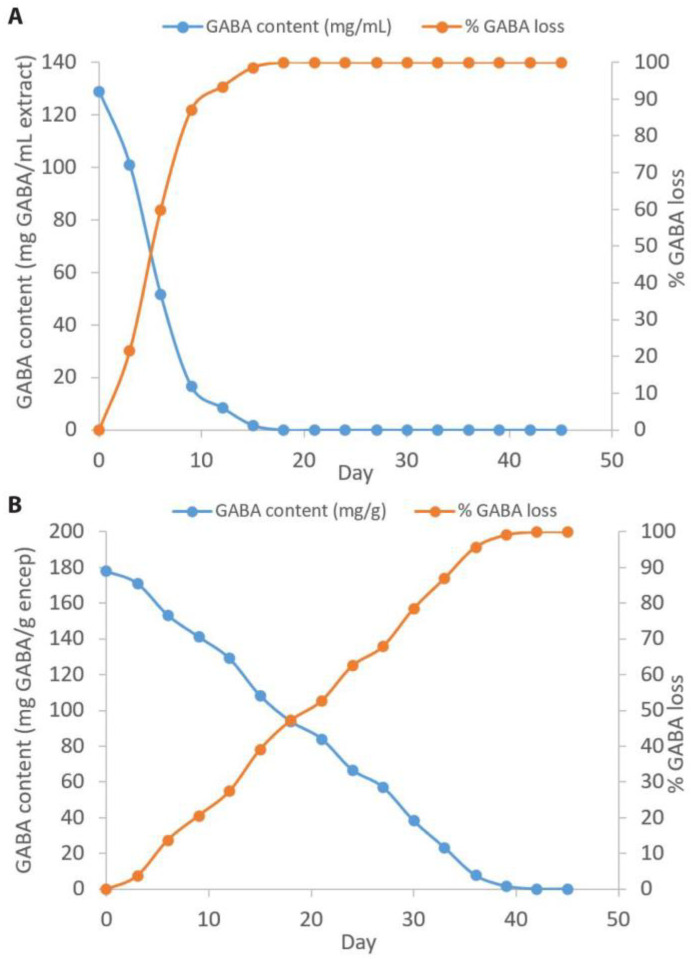
GABA content (mg) and GABA loss (%) of GABA extract from germinated brown rice (**A**) and encapsulated GABA with GA:MD as 2:2 as the wall material ratio (**B**).

The encapsulated GABA powder made by a ratio of wall material (GM and MD) as 2:2 and germinated brown rice extract (control) were separately stored in clear glass with a screw cap at 35 °C for 45 days (Figure 3). The GABA content and %GABA loss were measured every three days. The initial GABA content in germinated brown rice extract was 129.08 mg GABA/mL extract and decreased to 1.82 mg GABA/mL extract on day 15, which refers to GABA loss as 98.59%. After that, the GABA content could not be detected on day 18 onwards. In the case of encapsulated GABA, the initial GABA content was found to be 178.05 mg GABA/g encapsulated powder and decreased to 108.45 mg GABA/g encapsulated powder on day 15, referring to GABA loss as 39.09%. The GABA content of encapsulated GABA could not be detected on day 42 and beyond. Statistical analysis using independent t-tests showed that %GABA loss of the encapsulated GABA was significantly lower than the non-encapsulated one from day 3 and afterward.

In the case of encapsulated GABA, using the freeze-drying encapsulation technique, the GABA extract acted as a core molecule coated with the mixture of GA and MD used as wall material. The wall material acted as a barrier to protect the active compound in the core molecule from the environment, such as temperature, oxygen, pH, and light [8,9,10]. Moreover, the properties of MD and GA included film-forming capacity, which created a barrier between the GABA compound and oxygen and temperature [11,12]. The results of this study indicate that the GABA extract can be kept in clear glass with a screw cap at 35 °C for 18 days, but encapsulated GABA can prolong life until 42 days, which was higher than GABA extract at 3.25 times.

## 3. Materials and Methods

### 3.1. Raw Material Preparation and Chemicals

KDML 105 paddy rice was purchased from a farmer in Maha Sarakham province. The paddy rice was one round polished into brown rice. The brown rice was germinated to brown by soaking it in water for 24 h, wrapping it in a moist white cloth, and keeping it in the dark for two days. The germinated brown rice was dried with a tray dryer at 70 °C for 30 min and ground to powder. The sample was kept in a polyethylene plastic zip-lock bag at room temperature (25 °C) until further experiment. All the chemicals used were analytical grade. ɣ-aminobutyric acid, sodium hydroxide, phenol crystalline, and ethanol were purchased from Sigma-Aldrich (St. Louis, MO, USA). Gum arabic, maltodextrin (dextrose equivalent of 10), sodium hypochlorite reagent, and boric acid were obtained from CLS Supplier Co. Ltd. (Khon Kaen, Thailand).

### 3.2. GABA Extraction

GABA was extracted from the powder of germinated brown rice (5 g) in 80% ethanol (20 mL) and incubated at room temperature (25 °C) for 10 min at 200 rpm with a magnetic stirrer. The suspension was centrifuged at 5000× *g* for 10 min at 25 °C. The sediment was washed with 80% ethanol and centrifuged at 5000× *g* for 10 min at 25 °C. The washing step was repeated one time. All supernatant was pooled and evaporated by an evaporator at 60 °C until the final volume was 5 mL. The solution was kept in an opaque bottle at 4 °C until further experiment.

### 3.3. Preparation of Encapsulated Powders

The encapsulated powder was prepared according to Rezende and co-workers’ method with a few modifications [18]. The mixture of GA and MD (12 g) in the ratio 0:4, 1:3, 2:2, 3:1, and 4:0 (GA:MD, *w*/*w*) was soluble in 30 mL of water to prepare the dispersions. The GABA extract (10 mL) was added to the dispersions. The solution was continuously mixed for 30 min at room temperature (25 °C) at 200 rpm with a magnetic stirrer. The dispersion was frozen in the freezer at −20 °C for 24 h. The samples were placed in a freeze dryer and dried at −58.8 °C, with a pressure of 6.11 mbar and a vacuum of 0.42 mbar for 24 h. After freeze-drying, the samples were triturated using a mortar and pestle. The sample was kept in a polyethylene plastic zip-lock bag at room temperature (25 °C) until further experiment.

### 3.4. Determination of GABA Content

GABA content was determined according to Tanamool and co-worker’s method [1], with a few modifications. Briefly, 0.25 mL of borate buffer (0.2 M) was mixed with 1 mL of phenol reagent (6%), and then 0.1 mL of GABA extract was added to those mixers. Afterward, 0.4 mL of sodium hypochlorite (12.5%) was added and boiled for 10 min. The sample was cooled for 5 min, and the optical density was measured at 630 nm. The calibration curve of standard GABA was prepared with the range concentration of 0.02–0.625 mg, giving the equation Y = 208.51x + 0.0102, R^2^ = 0.9881, and used to determine the concentration of GABA in the sample.

### 3.5. Determination of Antioxidant Capacity

The antioxidant capacity was determined using a 2,2-diphenyl-1-picrylhydrazyl (DPPH) radicals assay, according to the method of Sripakdee and co-workers [29]. Briefly, the DPPH• solution in methanol (0.1 M) was prepared daily, and 3 mL of this solution was mixed with 1 mL of methanolic solutions of samples. The samples were incubated for 30 min in the dark at room temperature, and then the absorbance of the solution was measured at 517 nm. A blank sample containing 1 mL of methanol in the DPPH• solution was prepared daily, and its absorbance was measured (Ac). The experiment was carried out in triplicate. The percentage inhibition was calculated according to Equation (1).

Inhibition (%) = [(Ac − As)/Ac] ×100(1)

where Ac is the absorbance of the blank sample and As is the absorbance of the samples. The antioxidant activity of the GABA extract was expressed as 50% inhibitory concentration (IC50), which represents the sample content (mL) required to inhibit 50% of the free radical-antioxidant activity. The antioxidant activity of encapsulated powders was expressed as IC50, meaning the sample content (g) required to inhibit 50% of the free radical-antioxidant activity.

### 3.6. Determination of Encapsulation Efficiency

The encapsulation efficiency was determined according to the method of Oliyaei and co-workers with a few modifications [13]. The encapsulation efficiency was calculated from the ratio between the final content of GABA in all of the obtained encapsulation powder and the content of GABA in GABA extract (10 mL) added to the produced encapsulation powder. The % encapsulation efficiency was calculated according to Equation (2).

(2) $$\mathrm{Encapsulation}\;\mathrm{efficiency}\;(\%\mathrm{EE})=\frac{GABA\;content\;in\;total\;encapsulation\;powder\;(mg)}{GABA\;content\;in\;10\;mL\;of\;GABA\;extract\;(mg)}\;\times \;100$$

### 3.7. Process Yield Determination

The process yield was determined according to the method of Borompichaichartkul and co-workers with a few modifications [30]. The process yield was determined by the ratio of the mass of powders collected to the total mass of solids in the feed. The % process yield was calculated according to Equation (3).

(3) $$\mathrm{Process}\;\mathrm{yield}\;(\%)=\frac{Weight\;of\;dried\;powder\;(g)}{Weight\;of\;initial\;solid\;before\;drying\;(g)}\;\times \;100$$

### 3.8. Moisture Content and Water Activity Measurements

The moisture content of the samples was calculated from the weight loss after heating the sample in a drying oven at 105 °C until a constant was obtained [19]. The % moisture content was calculated according to Equation (4).

(4) $$\mathrm{Moisture}\;\mathrm{content}\;(\%)=\frac{Initial\;weight\;of\;sample\;-\;final\;weight\;of\;sample\;(g)}{Initial\;weight\;of\;sample\;(g)}\;\times \;100$$

### 3.9. Solubility Measurement

Solubility was determined according to the method proposed by Pan-utai and Iamtham with a few modifications [10]. One gram of GABA encapsulated was mixed with 50 mL of distilled water, and the mixture was stirred with a magnetic stirrer for 30 min. The suspension was centrifuged at 3000× *g* for 5 min. An aliquot of the supernatant was transferred to a pre-weighed aluminum can and dried at 105 °C for five hours with an air oven until there was a constant mass. The solubility was calculated by weight difference and expressed in percentage (%) according to Equation (5).

(5) $$\mathrm{Solubility}\;(\%)=\frac{Final\;weight\;of\;sample\;after\;drying\;(g)}{Initial\;weight\;of\;sample\;(g)}\;\times \;100$$

### 3.10. Bulk Density and Flowability Measurements

Bulk density was determined according to the method proposed by Pandey and Mishra with a few modifications [3]. The bulk density was reported as tapped bulk density. In detail, 2 g of GABA encapsulated was poured into a 25 mL graduated cylinder, tapping the cylinder until the constant volume was reached, and then calculated using Equation (6). Loose bulk density was also calculated by adding 2 g of GABA encapsulated into a 25 mL graduated cylinder but un-tapping and then using Equation (7) to calculate flowability in the following method.

(6) $$\mathrm{Tapped}\;\mathrm{bulk}\;\mathrm{density}\;(g/\mathrm{mL})=\frac{Weight\;of\;sample\;(g)}{Volume\;of\;sample\;after\;tapped\;(mL)}$$

(7) $$\mathrm{Loose}\;\mathrm{bulk}\;\mathrm{density}\;(g/\mathrm{mL})=\frac{Weight\;of\;sample\;(g)}{Volume\;of\;sample\;(mL)}$$

Flowability was determined according to the method of Pandey and Mishra [3], which was calculated in terms of the Hausner ratio (Equation (8)) and Carr’s Index (Equation (9)). They were calculated using the value of loose bulk density and tapped bulk density from the bulk density method. The flowability was determined by using data in Table 5.

(8) $$\mathrm{Hausner}\;\mathrm{Ratio}\;(\mathrm{HR})=\frac{Tapped\;bulk\;density\;}{Loose\;bulk\;density}$$

(9) $$\mathrm{Loose}\;\mathrm{bulk}\;\mathrm{density}\;(g/\mathrm{mL})=\frac{Tapped\;bulk\;density\;-\;Loose\;bulk\;density}{Tapped\;bulk\;density}$$

### 3.11. Morphology and Stability of the Encapsulated GABA Powder Analyses

The morphology of GABA encapsulated powder produced with different encapsulation agents was observed with a field electric scanning electron microscope (SEM, Tescan MIRA, Brno, Czech Republic). Samples were placed in a carbon support and coated with a layer of gold. Images with magnifications of 1 kx and 3 kx were recorded at the maximum voltage of 30 kV. The encapsulated GABA powder, which was made by a ratio of wall material (GM and MD) as 2:2 and the GABA extract, was separately stored in clear glass with a screw cap at 35 °C for 45 days. The GABA content and GABA loss (%) were measured every 3 days.

### 3.12. Statistical Analysis

Data were analyzed using SPSS 16.0 software with triplicate data. One-way analysis of variance (ANOVA) and Duncan’s multiple comparison tests were performed to determine the significant difference (*p* < 0.05) between the encapsulated samples. Duncan’s test is more sensitive than other tests (such as Tukey’s or Bonferroni’s). It has more statistical power to detect differences between group means because it uses a stepwise procedure to control for type I errors [31].

## 4. Conclusions

In summary, the study investigated the effects of using gum arabic (GA) and maltodextrin (MD) to encapsulate gamma-aminobutyric acid (GABA) derived from brown rice through a freeze-drying technique. It assessed different ratios of GA and MD to determine their influence on the properties of the encapsulated GABA powder. The findings indicate that a 2:2 ratio of GA to MD yields the highest GABA content and encapsulation efficiency, resulting in a powder with improved stability and a unique structure. The study concludes that this method effectively preserves GABA extracts from brown rice while enhancing their beneficial properties.

## Figures and Tables

**Table 1:** GABA content of KDML 105 brown rice, KDML 105 germinated brown rice, and germinated brown rice extract.

Sample	GABA Content
KDML 105 brown rice (mg/g DW)	13.08 ± 0.05 ^a^
KDML 105 germinated brown rice (mg/g DW)	30.24 ± 0.10 ^b^
Germinated brown rice extract (mg/mL)	129.12 ± 1.83 ^c^

Different letters indicate that the values with significant differences (*p* < 0.05).

**Table 2:** GABA content and antioxidant activity of encapsulated GABA with different wall material ratios (GA:MD).

Wall Material (GA:MD, *w*/*w*)	GABA Content of Encapsulated GABA (mg/g DW)	DPPH (IC50)
0:4	54.75 ± 1.38 ^e^	1.09 ± 0.49 ^a^
1:3	71.15 ± 1.16 ^c^	1.41 ± 0.51 ^a^
2:2	90.77 ± 1.90 ^a^	1.80 ± 0.29 ^a^
3:1	81.82 ± 1.34 ^b^	1.63 ± 0.53 ^a^
4:0	68.34 ± 1.58 ^d^	1.36 ± 0.73 ^a^

Different letters indicate that the values have significant differences (*p* ≤ 0.05).

**Table 3:** Physical properties of encapsulated GABA with different wall material ratios (GA:MD).

Wall Material (GA:MD, *w*/*w*)	Moisture Content (%)	Water Activity (aw)	Solubility (%)	Bulk Density (g/mL)
0:4	6.40 ± 0.46 ^a^	0.58 ± 0.05 ^a^	84.11 ± 4.83 ^c^	0.48 ± 0.04 ^a^
1:3	5.48 ± 0.82 ^b^	0.44 ± 0.11 ^a^	84.56 ± 1.64 ^b^	0.49 ± 0.05 ^a^
2:2	5.06 ± 0.74 ^bc^	0.42 ± 0.13 ^b^	90.62 ± 6.84 ^a^	0.47 ± 0.05 ^a^
3:1	4.54 ± 0.85 ^c^	0.38 ± 0.04 ^a^	80.36 ± 4.28 ^c^	0.49 ± 0.03 ^a^
4:0	3.79 ± 0.59 ^d^	0.36 ± 0.11 ^a^	81.24 ± 4.22 ^b^	0.50 ± 0.03 ^a^

Different letters indicate the values with significant differences (*p* ≤ 0.05).

**Table 4:** Hausner Ratio, Carr’s index, and flowability of encapsulated GABA.

Wall Material (GA:MD, *w*/*w*)	Carr’s Index (CI)	Hausner Ratio (HR)	Flowability
0:4	44.56 ± 4.97 ^c^	1.82 ± 0.16 ^d^	Very very poor
1:3	41.02 ± 4.21 ^c^	1.70 ± 0.13 ^cd^	Very very poor
2:2	36.72 ± 4.85 ^b^	1.59 ± 0.12 ^bc^	Very poor
3:1	35.14 ± 3.96 ^b^	1.55 ± 0.96 ^b^	Very poor
4:0	29.72 ± 4.40 ^a^	1.42 ± 0.09 ^a^	poor

Different letters were indicated that the values with significant differences (*p* ≤ 0.05).

**Table 5:** Specification for Carr’s index and Hausner ratio [24].

Flowability	Carr’s Index	Hausner Ratio
Excellent	0–10	1.00–1.11
Good	11–15	1.12–1.18
Fair	16–20	1.19–1.25
Possible	21–25	1.26–1.34
Poor	26–30	1.35–1.45
Very poor	32–37	1.46–1.59
Very, very poor	>38	>1.60

## Data Availability

The data presented in this study are available on request from the corresponding author.
